# Data set from chemical sensor array exposed to turbulent gas mixtures

**DOI:** 10.1016/j.dib.2015.02.022

**Published:** 2015-03-20

**Authors:** Jordi Fonollosa, Irene Rodríguez-Luján, Marco Trincavelli, Ramón Huerta

**Affiliations:** University of California, San Diego, USA

**Keywords:** Chemometrics, Machine olfaction, Electronic nose, Chemical Sensing, Machine learning

## Abstract

A chemical detection platform composed of 8 chemo-resistive gas sensors was exposed to turbulent gas mixtures generated naturally in a wind tunnel. The acquired time series of the sensors are provided. The experimental setup was designed to test gas sensors in realistic environments. Traditionally, chemical detection systems based on chemo-resistive sensors include a gas chamber to control the sample air flow and minimize turbulence. Instead, we utilized a wind tunnel with two independent gas sources that generate two gas plumes. The plumes get naturally mixed along a turbulent flow and reproduce the gas concentration fluctuations observed in natural environments. Hence, the gas sensors can capture the spatio-temporal information contained in the gas plumes. The sensor array was exposed to binary mixtures of ethylene with either methane or carbon monoxide. Volatiles were released at four different rates to induce different concentration levels in the vicinity of the sensor array. Each configuration was repeated 6 times, for a total of 180 measurements. The data is related to “Chemical Discrimination in Turbulent Gas Mixtures with MOX Sensors Validated by Gas Chromatography-Mass Spectrometry”, by Fonollosa et al. [1].

The dataset can be accessed publicly at the UCI repository upon citation of [1]: http://archive.ics.uci.edu/ml/datasets/Gas+senso+rarray+exposed+to+turbulent+gas+mixtures.

**Specifications Table**

**Table:** 

Subject area	*Chemistry*
More specific subject area	*Chemometrics, Machine Olfaction, Electronic Nose, Chemical Sensing, Machine Learning*
Type of data	*Text Files*
How data was acquired	*Metal Oxide (MOX) gas sensors provided by Figaro Inc. placed in a turbulent wind tunnel. Temperature and RH were recorded continuously with SHT15 sensor (Sensirion).*
Data format	*Raw data. Time-series.*
Experimental factors	*For each measurement 8 time series corresponding to MOX sensors׳ conductivity are provided. Temperature and humidity are provided in additional time series.*
Experimental features	*Sensors were exposed to clean air before and after sample presentation to acquire rising/decay transient portions of the signals.*
Data source location	*San Diego, California, US.*
Data accessibility	*Data in public repository:*
	http://archive.ics.uci.edu/ml/datasets/Gas+sensor+array+exposed+to+turbulent+gas+mixtures
	*Citation of*[1]*is required.*

**Value of the data**

• Realistic scenario: Sensors sampling in turbulent environment, in which generated gas plumes were mixed naturally along a wind tunnel, creating fluctuations in the gas concentrations.
• Complete time series are provided, including baseline, rising/decay portion, and steady state. System sensitive to gas turbulence.
• Dataset generated from chemical sensors exposed to three different volatiles, each volatile presented at different concentration levels. The problem can be formulated either as a classification problem to determine which gas is present or as a regression task to determine the gas concentration levels.
• Concentration levels validated by means of gas chromatography analysis.
• Dataset suitable for the benchmark of different Machine Learning techniques for chemical sensing.

## 1 Experimental design, materials and methods

### 1.1 Experimental setup

#### 1.1.1 Chemical detection platform

The spatio-temporal structure of gas plumes in outdoor environments is mainly determined by turbulent diffusion rather than molecular diffusion. Hence, when a volatile is emitted from its source, the released molecules are carried in the direction of the fluid flow, forming a patchy plume in the downstream direction and decreasing the mean concentration as the volatile molecules spread out. Since, in open environments, air direction and intensity change in time, generated gas plumes have complex, irregular, shifting structures [2]. Similarly, when sources of different volatiles are present, the concentration of the compounds changes dynamically in time and space, generating non-uniform gas mixtures.

We designed a general purpose chemical sensing platform that included eight commercialized metal oxide gas sensors (provided by Figaro Inc.) to detect analytes and follow the changes of their concentration in a wind tunnel facility. The sensor׳s response magnitude to the chemical analyte is signaled by a change in the electrical conductivity of the sensor׳s film. Hence, changes in the analyte concentration (mostly due to patches and eddies in the chemical plume) are reflected in the sensor׳s response in real-time and are the origin of the temporal resolution (i.e. fluctuations in the time series). Table 1 shows the models and number of units included in the sensor array. The operating temperature of the sensors is controlled by the voltage applied to the built-in sensors׳ heaters. The voltage on the heaters was kept constant at 5 V.

The generated chemical sensors׳ time series, along with temperature and relative humidity readings provided by SHT15 sensor system (Sensirion), were acquired at a sampling rate of 20 ms for the whole duration of the experiment.

#### 1.1.2 Wind tunnel

**Fig. 1:**
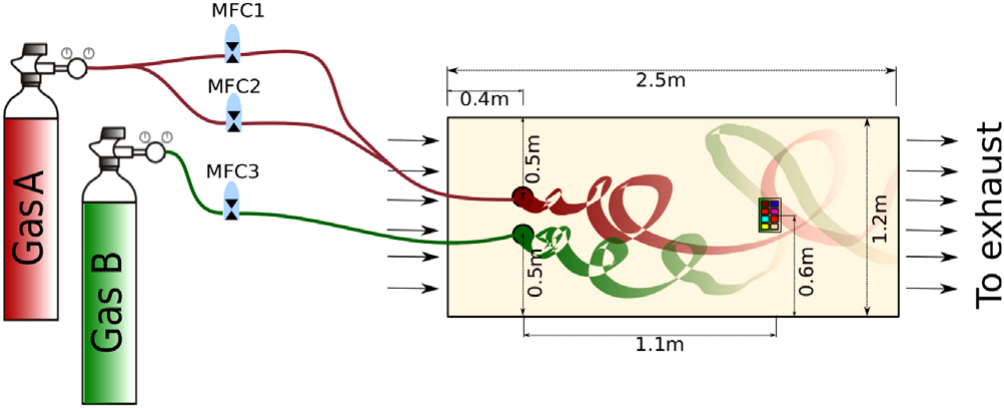
The wind tunnel includes two gas sources that generate two independent gas plumes. The facility was used to collect sensors׳ responses when placed in a turbulent environment.

We constructed a 2.5 m×1.2 m×0.4 m wind tunnel (see Fig. 1), a research test-bed facility endowed with a computer-supervised mass flow controller system. The resulting wind tunnel operates in a propulsion open-cycle mode, by continuously drawing external turbulent air throughout the tunnel and exhausting it back to the outside, thereby creating a relatively less-turbulent airflow moving downstream towards the end of the test field. The wind tunnel included two gas sources. The gas concentration of each plume was controlled by a set of mass flow controllers (MFCs). Each source was controlled independently to release the selected volatiles at different flow rates, which induced different concentration levels in the sensors׳ location. The wind generator created a turbulent flow that constantly displaced the introduced volatiles towards the exhaust outlet.

To control airflow in the wind tunnel, we utilize a multiple-step motor-driven exhaust fan located at the outlet of the test section. The wind speed is controlled by a multiple-step motor-driven exhaust fan that rotates at a constant frequency (3900 rpm), generating a turbulent flow that can be characterized by the mean speed at the axis of the wind tunnel. The wind speed was selected from previous study to generate gas plumes that interact each other. The estimated wind speed at the main axis of the wind tunnel was 0.21±0.005 m/s [3].

### 1.2 Methods

We exposed the detection unit to mixtures of ethylene with methane or carbon monoxide in the wind tunnel. The considered volatiles were provided in mixtures of medical dry air, at certified concentrations of 2500 ppm, 1000 ppm and 4000 ppm for ethylene, methane, and carbon monoxide, respectively. The mixtures were created by releasing ethylene at one source, whereas the interfering plumes were originated releasing methane or carbon monoxide at the interfering source. Hence, ethylene and methane/carbon monoxide were consistently released from the same source.

The gases were released at different flow rates. Although the gas concentration at the gas sources was constant for a given volatile, it is important to note that different gas flow rates at the gas sources resulted in plumes of different gas concentrations. Hence, gas sensors showed higher responses to plumes generated at higher flow rates. In order to estimate the actual concentration at which the detection unit was exposed, we used a GC–MS system as a gold standard. In particular, we utilized a Trace GC ultra coupled with ISQ single quadrupole MS (Themo Scientific) with a 30 m length, 0.32 mm diameter a GS-GASPRO chromatogram column (Agilent). It is important to note that due to the experimental protocol, the obtained mass spectra were the result of the averaged gas sample acquired during the whole sampling time. Therefore, the fluctuations in the gas concentration levels due to air turbulence were not expected to be reproduced by means of the GC–MS analysis, but the chromatogram analysis provides a quantification of the number of particles captured during the sampling time.

To generate the experimental dataset, we released each volatile at four different flows. The complete dataset was composed of 180 measurements, which were performed in a random order. Table 2 shows the number of repetitions performed for each experimental configuration.

**Fig. 2:**
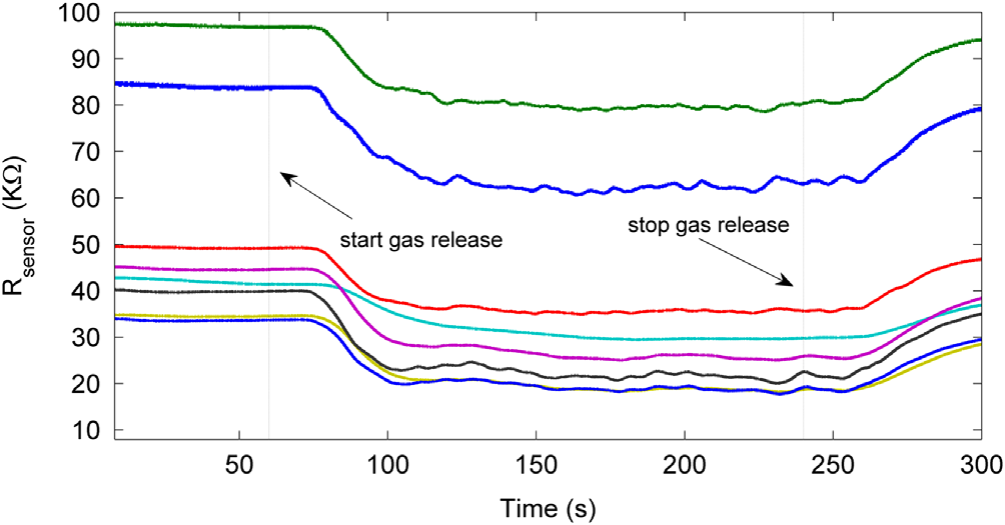
Signals acquired from the sensor unit when being exposed to a turbulent mixture of ethylene and CO. The detection platform is sensitive to gas turbulence present in the wind tunnel.

The total duration of each measurement was 300 s. During the first 60 s, no gas was released at the gas sources. At *t*=60 s, both sources started to release the corresponding volatile at the specified flow rate. The duration of the gas release was 180 s. Finally, the system acquired the recovery to the baseline for another 60 s. During the whole duration of the experiment, the sensors׳ signals were acquired constantly every 20 ms, generating 8 time series that were indicative of the gas conditions presented to the sensors. Fig. 2 shows an example of the acquired time series for a mixture of ethylene with carbon monoxide.

Finally, it is important to note there is no symmetry in the spatial distribution of the plume with respect to the main axis (i.e., the line connecting the chemical analyte source to the exhaust). A plume demonstrating a perfect symmetry in real environmental conditions is rare due to the existent non-symmetry of the volume enclosing the field, the inhomogeneous temperature in the ambient, and the variability of the flow direction.

### 1.3 Attribute description

The dataset is presented in 180 text files, where each file corresponds to a different measurement. The filenames identify the measurements as follows: The first 3 characters of the filename are a local identifier, which is not related to the order of the measurements; characters 5–8 indicate the concentration level of Ethylene released at source2 (n: zero, L: Low, M: Medium, H: High); the last 4 characters indicate the gas released at source1 (Me: Methane, CO: Carbon Monoxide) and the concentration level. For example, file 007_Et_L_Me_H contains time series acquired when Ethylene was released at Low concentration (31 ppm, mean concentration) and Methane at High concentration (131 ppm, mean concentration).

Each file includes the acquired time series, presented in 11 columns: Time (s), Temperature (°C), Relative Humidity (%), and the readings of the 8 gas sensors: TGS2600, TGS2602, TGS2602, TGS2620, TGS2612, TGS2620, TGS2611, TGS2610. The readings can be converted to sensor resistance by Rs(KOhm)=10^⁎^(3110−A)/A, where A is the acquired value.

## Figures and Tables

**Table 1:** MOX sensors included in the 8-sensor array. The manufacturer (Figaro Inc.) adapts the sensing layer to detect different target gases.

Sensor type	Number of units
TGS2611	1
TGS2612	1
TGS2610	1
TGS2600	1
TGS2602	2
TGS2620	2

**Table 2:** Sample distribution in the dataset along with rate of chemical release (in sccm) and corresponding induced gas concentration level (in ppm) in the vicinity of the sensors. 180 measurements were performed in total.

			Ethylene@2500 ppm			
			20 sccm	14 sccm	8 sccm	0 sccm
			96 ppm	46 ppm	31 ppm	0 ppm
CO@4000 ppm	200 sccm	460 ppm	6	6	6	6
	140 sccm	397 ppm	6	6	6	6
	800 sccm	270 ppm	6	6	6	6
	0 sccm	0 ppm	6	6	6	–

Methane @1000 ppm	300 sccm	131 ppm	6	6	6	6
	200 sccm	115 ppm	6	6	6	6
	100 sccm	51 ppm	6	6	6	6
	0 sccm	0 ppm	6	6	6	–
